# Heparanase Interacts with Resistin and Augments Its Activity

**DOI:** 10.1371/journal.pone.0085944

**Published:** 2014-01-21

**Authors:** Daniela Novick, Sara Barak, Neta Ilan, Israel Vlodavsky

**Affiliations:** 1 Department of Molecular Genetics, The Weizmann Institute of Science, Rehovot, Israel; 2 Cancer and Vascular Biology Research Center, The Rappaport Faculty of Medicine, Technion, Haifa, Israel; Instituto Butantan, Brazil

## Abstract

In an attempt to isolate a heparanase receptor, postulated to mediate non-enzymatic functions of the heparanase protein, we utilized human urine collected from healthy volunteers. Affinity chromatography of this rich protein source on immobilized heparanase revealed resistin as a heparanase binding protein. Co-immunoprecipitation and ELISA further confirmed the interaction between heparanase and resistin. Importantly, we found that heparanase potentiates the bioactivity of resistin in its standard bioassay in which monocytic human leukemia cell line, THP1, differentiates into adherent macrophage-like foam cells. It is thus conceivable that this newly identified complex of heparanase and resistin exerts a stimulatory effect also in various inflammatory conditions known to be affected by the two proteins.

## Introduction

Heparan sulfate proteoglycans (HSPGs) are primary components at the interface between eukaryotic cells and the extracellular matrix (ECM) and are involved in modulating cell invasion and signaling loops that are critical for tumor growth, inflammation and kidney function [1]. Mammalian cells express primarily a single dominant functional heparanase enzyme, the sole heparan sulfate (HS) degrading endoglycosidase [2]–[4]. Enzymatic degradation of HS accomplishes removal of a vital cross linker of matrix proteins, release of growth factors and cytokines bound to HS, and release of oligosaccharides that can regulate protein–protein interaction. This leads to disassembly of the ECM and is therefore associated with tissue remodeling, inflammation, angiogenesis and metastasis [2]–[4]. Normally, heparanase is found mainly in platelets, mast cells, placental trophoblasts, keratinocytes and leukocytes. Heparanase released from activated platelets and cells of the immune system facilitates extravasation of inflammatory and tumor cells [5] and also stimulates endothelial mitogenesis, primarily through release of HS-bound growth factors (i.e., FGF, HGF, VEGF) residing in the ECM [6]. Both over-expression [7] and silencing [8] of the heparanase gene indicate that heparanase enhances cell dissemination and promotes the establishment of a vascular network that accelerates primary tumor growth and metastasis. Immunohistochemistry, in situ hybridization and real time-PCR analyses revealed that heparanase is up-regulated in essentially all major types of human cancer [3], [5], [9]. Heparanase exhibits also non-enzymatic activities, independent of its involvement in ECM degradation. It includes enhanced adhesion of various cancer cells [10], [11], enhanced Akt signaling and stimulation of PI3K- and p38-dependent endothelial cell migration [12], [13], Src mediated phosphorylation of the EGF receptor [14], phosphorylation of STAT [15], activation of TLR2 and 4 [16], and up regulation of VEGF [13] and HGF [17], all contributing to its potent pro-tumorigenic and pro-inflammatory activities [5]. The molecular mechanism by which heparanase elicits signal transduction has not been resolved but is thought to involve the heparanase C-terminus domain [18] and various heparanase binding protein(s)/receptor(s) [18]–[20]. In an attempt to isolate such a receptor, we utilized human urine as a source for shed and/or alternatively spliced protein(s) that may lead to a cell surface receptor, an approach that has been used successfully to identify several soluble cytokine receptors (i.e., IL-6R, IFN-γR, TBPI, TBPII, LDLR, IFNα/βR) [21], [22]. Affinity chromatography of human urine has unexpectedly revealed that resistin, a protein implicated in inflammation, is associating with heparanase. We provide evidence that heparanase physically interacts with resistin and augments its activity. The results uncover a potential route for heparanase function in cancer and inflammation.

## Materials and Methods

Urine was kindly provided by Serono (Geneva, Switzerland). The urine pool (250 liter) was collected anonymously in monasteries in Italy from menopausal nuns in the mid 1980 originally for the isolation of the fertility hormone, Pergonal, and was given as a gift.

Therefore, participants provide their verbal, but not written (urine was obtained anonymously), informed consent to participate in this study. The study was carried out according to the high ethical standard of Serono. However, due to the long time passed (over 25 years), we are unable to track the original documentation. The current research was not conducted outside of our country of residence.

### Heparanase purification

cMyc-tagged 65-kDa latent heparanase protein was purified from medium conditioned by heparanase infected CHO cells [16]. Briefly, cells were grown to confluence in low serum (2.5%). Cells were then grown under serum-free conditions for 24 hours; conditioned medium (∼1 L) was collected, filtered, and loaded (20 hours, 4°C) on a heparin column (Hi Trep FF Heparin column, Pharmacia) equilibrated with 20 mM phosphate buffer, pH 6.0. Following washes (15 column volumes), heparanase was eluted with a linear salt gradient (100 mol/L to 1.5 mol/L NaCl) in 20 mM phosphate buffer (pH 6.0) and 1 mmol/L dithiothreitol. Heparanase is eluted from the column at 0.7 to 0.8 mol/L NaCl and appears as a single, highly purified protein band by Coomassie blue staining [16]. Constitutively active heparanase (GS3) [23] was purified from the conditioned medium of transiently transfected SF9 insect cells applying a similar purification procedure.

### Reagents and Antibodies

A pure preparation of human recombinant resistin expressed in HEK 293 was a gift from Serono and was used in all assays. Human leptin expressed in E. coli was purchased from Peprotech (Rocky Hill, NJ, USA). The resistin and heparanase preparations were assayed for the presence of bacterial endotoxin using the gel clot technique (Limulus amebocyte lysate, LAL test) and were found to contain <100 pg/ml and <10 pg/ml endotoxin (LPS), respectively. Both preparations were further diluted in the THP1 cell assay so that the final concentration of LPS was at least 1000 fold lower than that reported to elicit monocyte differentiation into macrophages. PMA (phorbol 12-myristate 13-acetate) and Oil Red O were from Sigma (St. Louis, MI). Anti resistin antibodies were raised in rabbits injected with resistin expressed in E. coli. Anti heparanase polyclonal antibody #1453 has been described previously [13], [14]; Anti Myc-Tag monoclonal antibody 9E10 was purchased from Santa Cruz Biotechnology (Santa Cruz, CA).

### Cells

THP1 (human acute monocytic leukemia cell line) cells were obtained from ATCC and cultured in RPMI 1640 medium containing 4.5 g/L glucose, 2 mM L-glutamine, 10 mM HEPES, 1.5 g/L sodium bicarbonate, 1 mM sodium pyruvate and 10% FCS.

### Concentration of crude urinary proteins

Urine was kindly provided by Serono and was used successfully to isolate several soluble cytokine receptors [21], [22]. For concentration, urine (250 liter) was filtered on a Millipore HVLP membrane (pore size, 0.5 pm) using a Pellicon cassette system. The filtrate was concentrated by tangential ultrafiltration to a final volume of 500 ml, with the aid of a PTGC Millipore membrane (molecular weight cut off 10,000). The concentrate was dialyzed against PBS containing 0.02% sodium azide and 1 mM benzamidine (Sigma), aliquoted into portions, and frozen [22].

### Isolation of a urinary heparanase binding protein

Recombinant cMyc-heparanase was immobilized on Affigel-10 Sepharose beads according to the manufacturer's (BioRad, Richmond, CA) instructions. Crude urinary proteins, batches of 500 mL concentrated 500 fold, were passed over the column at 4°C. The column was washed with 250 mL phosphate buffer (pH 7.4) containing 0.65 M NaCl. Bound proteins were eluted in 1 mL fractions with 25 mM citric acid solution (pH 2.2) containing benzamidine (1 mM), and each fraction was immediately adjusted to neutral pH with 1M Na_2_CO_3_.

### Mass spectrometry

Elution fractions from the affinity chromatography were concentrated using Amicon Ultrafree-MC 10,000 NWML filter units (Millipore Corp. Bedford, MA), subjected to SDS-PAGE in non reducing conditions, and the protein bands were visualized by silver staining. The protein band corresponding to 18 kDa was excised from the gel, electro-eluted and digested with trypsin. The resulting tryptic digest was subjected to liquid-chromatography and tandem mass spectrometry (C-MS/MS) on Orbitrap (Thermo) mass spectrometer (Smoler Proteomics Center, Technion, Haifa, Israel). The mass spectrometry data was clustered and analyzed using the Sequest software searching against the human database.

### Immunoprecipitation and immunoblotting

cMyc-latent heparanase (5 µg) or active (GS3) heparanase (1 µg) were incubated with resistin (5 µg) in a total volume of 50 µl 0.1% BSA in PBS, for 1 h at room temperature. The indicated antibody was added for a further incubation of 2 h followed by 45 min incubation with Protein-G beads. Beads were washed twice with PBS containing 0.05% Tween 20 and once with PBS. Sample buffer containing DTT (25 mM) was added, the samples were centrifuged and the supernatants were resolved by SDS-PAGE and immunoblotting, essentially as described [13]–[15].

### ELISA

Microtiter 96-well ELISA plates (Maxisorb; Nunc A/S, Roskilde, Denmark) were coated (20 h, 4°C) with 100 µl of cMyc-heparanase (5 µg/ml), active heparanase (2 µg/ml), or resistin (5–10 µg/ml) in PBS. The plates were washed with PBS containing 0.05% Tween 20 (washing solution) and blocked (1.5 h, at RT) with BSA stock solution (10%; KPL, Geithersburg, MD,) diluted 1∶10 in water. BSA stock solution was diluted 1∶15 in water for all other steps. The tested samples (cMyc-heparanase, active heparanase, resistin; 100 µl) were added to the wells for 2 h at RT, followed by incubation (2 h at RT) with the relevant secondary antibody [rabbit anti resistin (1∶500), rabbit anti heparanase #1453 (4 µg/ml), or mouse anti cMyc (2 µg/ml)]. Goat-anti-rabbit or anti mouse horseradish peroxidase (HRP, Jackson ImmunoResearch Labs, 1∶10,000 in PBS, 100 µl/well) conjugate was then added for 45 min at RT and color was developed by the addition of TMB (3,3′,5,5′-tetramethylbenzidine) substrate, and the absorbance at 630 nm was determined by an ELISA reader. Plates were washed three times between each step. In competition assays, the analyte was incubated with the competitor agent (30 min at RT) prior to application on the coating layer of the ELISA.

### Surface plasmon resonance

Analyses were performed using a BIAcore 3000 instrument (GE Healthcare). cMyc latent heparanase (0.02 mg/mL) was immobilized on a single channel of a CM5 sensor chip according to the manufacturer's instructions. Aliquots of the peak elution fraction from the cMyc latent heparanase affinity column were passed over the chip at various concentrations and the dissociation constant was calculated using BIAevaluation software.

### Resistin bioassay

THP1 cells were seeded in 48 wells Costar plates (0.5×10^6^/well in 0.3 ml RPMI containing 10% FCS and 100 nM PMA) followed by the addition of resistin, or resistin preincubated for 10 min at RT with cMyc latent or active heparanase (0.1 ml RPMI containing 0.1% FCS) according to the experimental design. After 4 days, medium was replaced with RPMI containing 0.2% FCS (0.5 ml/well). Differentiation was followed under the microscope and images were taken on day 6. Cells were stained with Oil Red on day 6 or 7; Quantification was performed using the ImageJ program (NIH).

### Oil Red O staining

Medium was removed followed by a gentle wash with PBS and formalin (10% in PBS). Formalin (10% in PBS, 0.5 ml) was then added for at least 1 h at room temperature followed by a wash with isopropanol (60% in H_2_O). Wells were then dried completely and Oil Red freshly diluted from a 10% stock solution (see below) was added for 10–30 min. Cells were then washed 3 times with H_2_O and photographed. Working solution of Oil Red was prepared by mixing 6 parts of Oil Red stock solution with 4 parts of H_2_O and incubating for 20 min at room temperature followed by filtering through 0.22 µm filter. Quantification was performed using the ImageJ program (NIH) that among others is widely used for cell count, density and statistical analysis [24] (oil red staining) or Student t-test (ELISA). p≤0.05 was considered significant.

Each experiment was repeated 3 times with similar results and each data point represents the mean ±SE of triplicate wells.

## Results

### Isolation of heparanase binding protein from human urine by affinity chromatography

**Figure 1:**
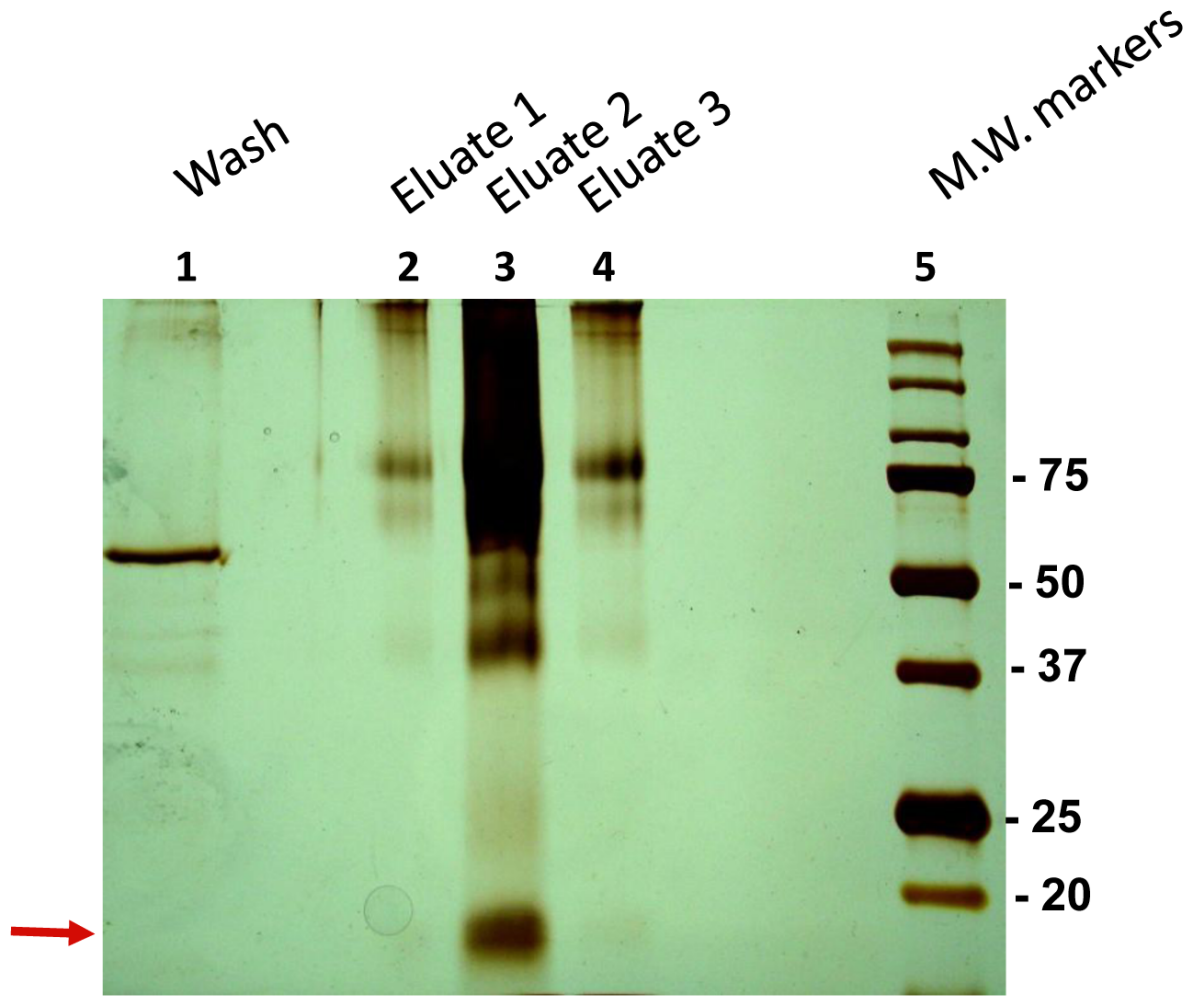
Silver staining (10% PAGE, non reducing conditions) of binding proteins eluted from affinity chromatography of human urine loaded on latent cMyc-heparanase coupled to sepharose beads. Lane 1, wash fraction. Lanes 2–4, elution fractions. Lane 5, molecular weight markers (in kDa). Resistin was identified in the 18 kDa band excised and subjected to MS analysis (arrow).

In an attempt to isolate a putative soluble heparanase receptor, concentrated urine was applied to a column comprised of cMyc-latent heparanase covalently immobilized to Sepharose beads. After extensive washing with high salt buffer, bound proteins were eluted at low pH. Aliquots of the various fractions were resolved by SDS-PAGE under non-reducing conditions and the protein bands were visualized by silver staining. Protein bands exhibiting molecular weight of 18 kDa, 25 kDa, 38 kDa and 75 kDa from lane #3 (Figure 1, elution 2) were submitted to liquid-chromatography and tandem mass spectrometry (MS) analysis. Most of the proteins eluted from the heparanase affinity chromatography and identified to sufficient confidence by at least three peptides, have been recognized in previous chromatographies/MS analyses and were thus considered non-specific (Table S1). In striking contrast, resistin (RETN) that was identified in the 18 kDa band by five peptides (R.GDLATCPR.G; R.AETTCHCQCAGMDWTGAR.C; R.AISSIGLECQSVTSR.G; K.TLCSMEEAINER.I; R.IQEVAGSLIFR.A) appeared specific and was never recognized in previous affinity chromatographies. Notably the correlation score for all resistin peptides was higher than 2 (2.2–3.85) and the delta correlation score was higher than 0.2 (0.44–0.65), pointing to a good peptide match with the protein (probability score = 2.67×10**^−10^**). We further confirmed the affinity of resistin to heparanase by additional methods described below.

### Heparanase co-immunoprecipitates with resistin

**Figure 2:**
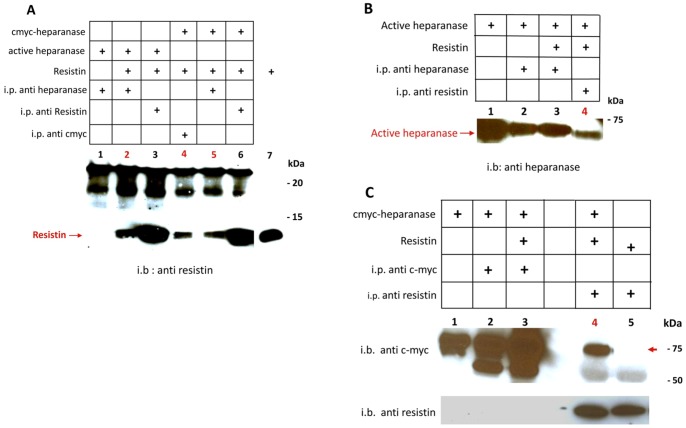
Co-immunoprecipitation of heparanase with resistin. **A**: Lanes 1–6: co-immunprecipitation of resistin with either latent cMyc-heparanase or active heparanase imunoprecipitated with the indicated antibodies. Lane 7: a reference resisitin without co-immunprecipitation was run on the same gel. Immunoblot detection with rabbit anti resistin. The co-immunoprecipitated resistin is indicated by an arrow. **B**: Lane 1: a reference active heparanase without co-immunprecipitation. Lanes 2–4: co-immunprecipitation of resistin with active heparanase imunoprecipitated with the indicated antibodies. Immunoblot detection with rabbit anti heparanase. The co-immunoprecipitated active heparanase is indicated by an arrow. i.p- immunprecipitation; i.b- immunoblotting. **C**: Lane 1: a reference latent cmyc- heparanase without co-immunprecipitation. Lanes 2–5: co-immunprecipitation of resistin with cmyc-latent heparanase imunoprecipitated with the indicated antibodies (all run on the same gel). Immunoblot detection with mouse anti cMyc monoclonal antibody. The co-immunoprecipitated latent cMyc-heparanase is indicated by a red arrow.

Samples of active (GS3; Figure 2A, lane 2) or latent cMyc-heparanase (Figure 2A, lanes 4 and 5) were incubated with recombinant resistin and immunoprecipitated with anti heparanase (Figure 2A lanes 2 & 5) or anti cMyc antibodies (Figure 2A, lane 4) followed by immunoblotting with anti resistin antibody. In both cases, resistin was co-immunoprecipitated with heparanase. Note that under reducing conditions resistin runs as a 12 kDa band (Figure 2A, lane 7, resistin directly loaded on the gel). The co-immunoprecipitation was also demonstrated in a reciprocal experiment, namely, immunoprecipitation with anti resistin antibodies followed by immunoblotting with anti heparanase antibodies (Figure 2B, lane 4). Next, we performed co-immmunoprecipitation of cMyc tagged latent heparanase with resistin followed by immunoblotting with anti cMyc (Figure 2C, upper panel) or anti resistin (Figure 2C, lower panel, lane 4) antibodies, further demonstrating the interaction between the two proteins.

### Heparanase interacts with resistin in a solid state

**Figure 3:**
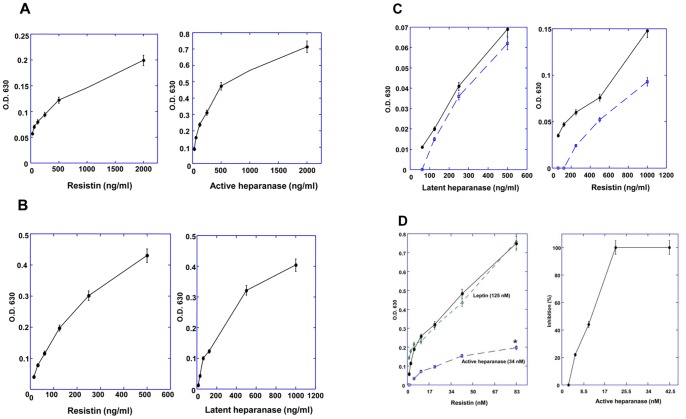
**A–C**: ELISA of resistin with either latent cMyc-heparanase or active heparanase. **A** (left panel): coating layer: active heparanase; analyte: resistin; detection: anti resistin antibody. **A** (right panel): coating layer: resistin; analyte: active heparanase; detection: anti heparanase antibody. **B** (left panel): coating layer: cMyc latent heparanase; analyte: resistin; detection: anti resistin antibody. **B** (right panel): coating layer: resistin; analyte: cMyc latent heparanase; detection: anti cMyc antibody. **C** (left panel): coating layer: resistin in the absence (solid line) or presence of DTT (broken line); analyte: cMyc latent heparanase; detection: anti cMyc antibody. **C** (right panel): coating layer: cMyc latent heparanase; analyte: resistin untreated (solid line) or treated with DTT (broken line); detection: anti resistin antibody. Reagent background (O.D 630) obtained under the ELISA conditions and consisting of all layers of the ELISA except the analyte, was subtracted from each data point. Each data point represents the mean ±SE of triplicate wells. **D** (left panel): Competition of resistin binding (analyte) to cMyc latent heparanase (coat layer) by active heparanase. Resistin binding was measured without competitor (full circles, solid line), or in the presence of active heparanase (34 nM, squares, broken line), or leptin, a negative control (125 nM, diamonds, broken line). *p = 0.01 for resistin binding in the absence or presence of active heparanase. **D** (right panel): Competition on the binding of resistin (analyte, 200 ng/ml) to cMyc latent heparanase (coat layer) by active heparanase, presented as percent inhibition. Bound resisitin was detected by anti resistin antibodies. Reagent background (nonspecific signals, O.D 630) consisting of all layers of the ELISA except the analyte, was subtracted from each data point. Each data point represents the mean ±SE of triplicate wells.

**Figure 4:**
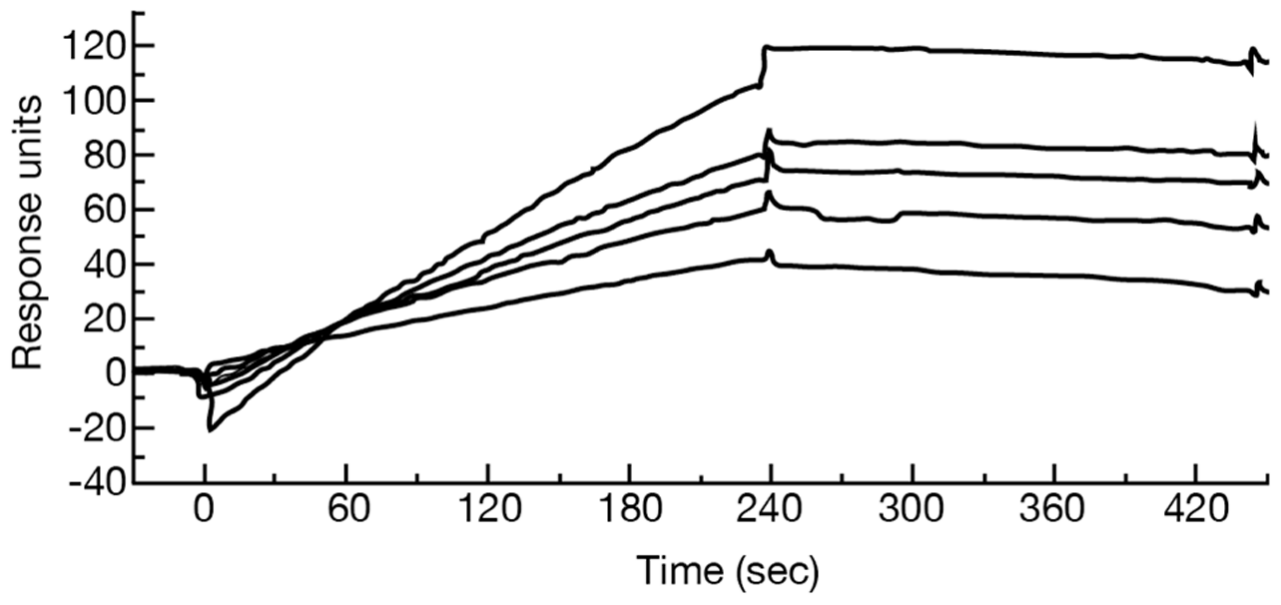
Surface plasmon resonance analysis of the peak affinity chromatography eluate binding to immobilized latent cMyc latent heparanase. Shown are binding curves (top to bottom) of the following estimated resistin concentrations: 10, 6.25, 5, 3.3 and 2.5 nM.

The interaction of heparanase with resistin was further substantiated by ELISA experiments. Microtiter plates were coated with active heparanase, resistin, or latent cMyc-tagged heparanase and the analytes were tested for interaction with the coating agent using a corresponding detecting antibody. As demonstrated in Figure 3, resistin was found to interact with heparanase whether used for coating or as an analyte (Figure 3A, B). Both active (Figure 3A) and cMyc-tagged latent heparanase (Figure 3B) reacted with resistin in a nanogram range. Leptin served as a negative control and did not exhibit such an interaction. To test whether the interaction of resistin with heparanase depends on its dimeric or multimeric form (S-S bonds) the ELISA was performed in the presence of excess (2000 fold) reducing agent (0.1 mM DTT). As shown in Figure 3C, reducing conditions did not affect the interaction between heparanase and resistin. We have also performed competitive ELISA (Figure 3D) and demonstrated that resistin binding to cMyc-latent heparanase (the coating layer) is inhibited following preincubation of the analyte (resistin, 200 ng/ml) with increasing concentrations of active heparanase, yielding complete inhibition at 20 nM (∼1 µg/ml; p = 0.01). In contrast, leptin did not compete for resistin binding to heparanase (Fig. 3D, Leptin). Surface plasmon resonance analysis in which the eluate was passed on cmyc latent heparanase coupled to a BIAcore chip, revealed a classical binding curve and a calculated KD of 3.92×10^−10^ (Figure 4).

### Heparanase modulates resistin bioactivity in human cells

**Figure 5:**
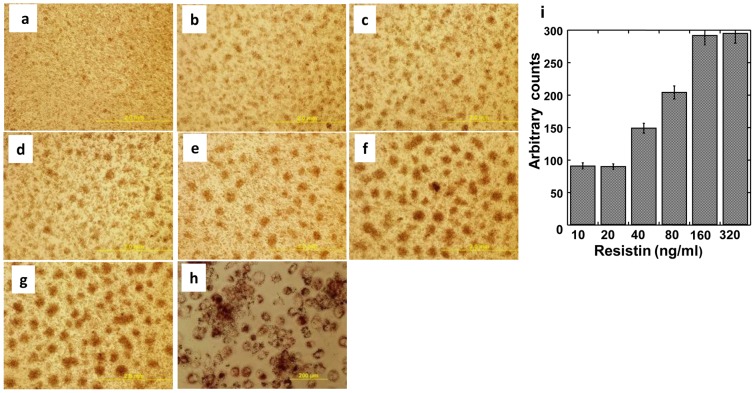
Dose response of resistin mediated differentiation of human THP1 cells. Cells were treated with PMA (100 nM, **a**) or PMA with 10 (**b**), 20 (**c**), 40 (**d**), 80 (**e**), 160 (**f**) and 320 (**g**) ng/ml resistin. **h**. Oil red staining of the differentiated cells at the highest (320 ng/ml) concentration of resistin. Each experiment was repeated 3 times yielding similar results. A representative experiment is presented. **i**. Quantification of cell differentiation (oil red staining) in response to increased resistin concentrations. Each data point represents the mean ±SE of triplicate wells. *p = 0.001 for 20 vs. 160 ng/ml resistin.

**Figure 6:**
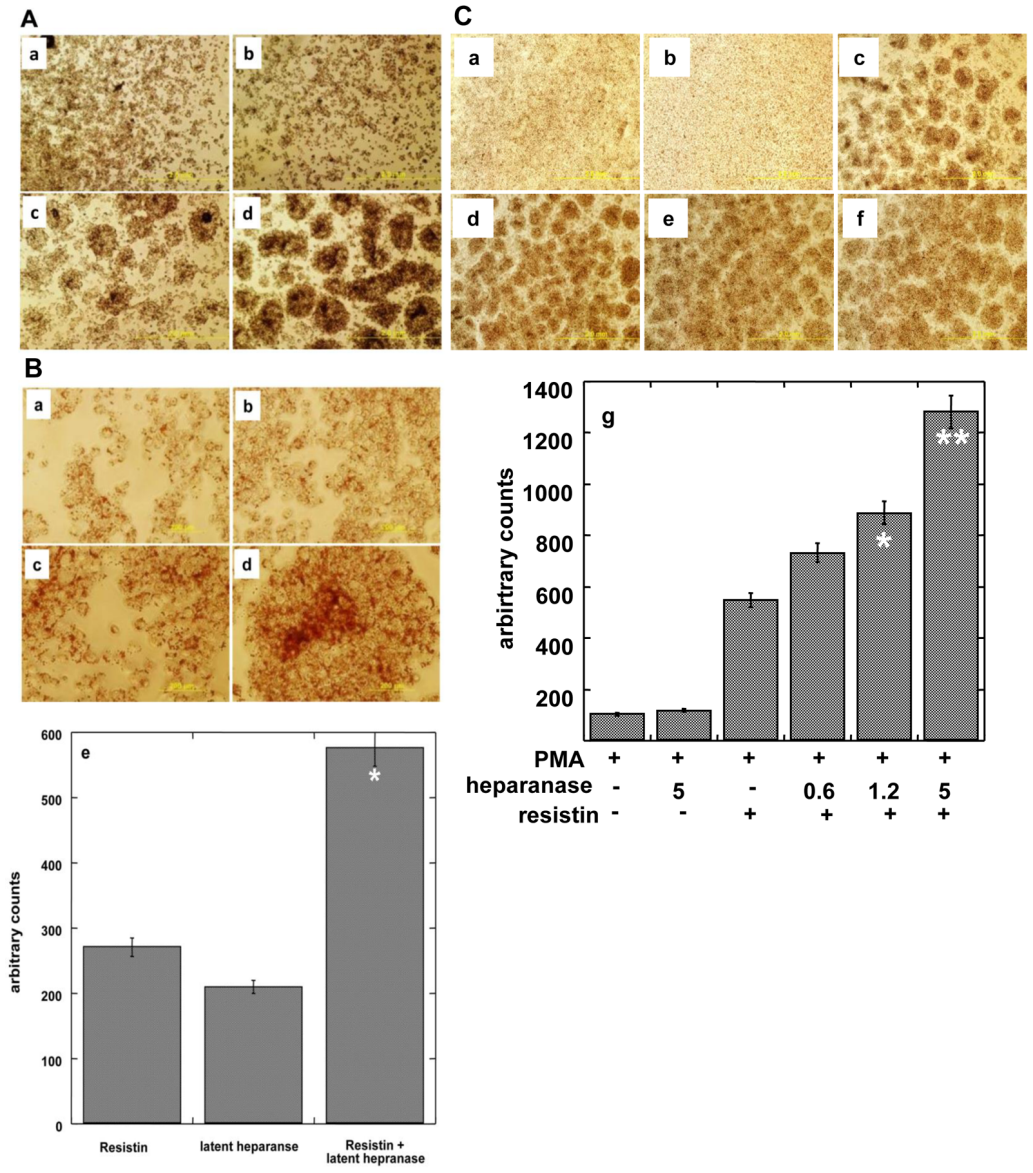
**A, B**: Enhancement of resistin (0.3 µg/ml) and PMA (100 nM) mediated differentiation of THP1 cells by latent cMyc-heparanase (1.25 µg/ml). Cells were treated with PMA in the absence (**a**), or presence of latent heparanase (**b**), resistin (**c**) or both (**d**). Cells were then subjected to Oil red staining to visualize oil droplets. Shown are representative photomicrographs at low (×4, **A**) and high (×20, **B**) magnifications. Each experiment was repeated 3 times yielding similar results. A representative experiment is presented. **e**. Quantification of cell differentiation (oil red staining) in response to resistin, heparanase, or both. Each data point represents the mean ±SE of triplicate wells. Counts (80) obtained for control cells treated with PMA alone were subtracted from all the treatment conditions shown in panel **e**. *p = 0.001 for resistin vs. resistin+heparanase. **Figure 6**. **C**: Enhancement of resistin (50 ng/ml) and PMA (100 nM) mediated differentiation of THP1 cells by active heparanase. Dose response of heparanase is shown. Cells were treated with PMA in the absence (**a**) or presence of active heparanase (5 µg/ml; **b**), resistin (**c**), or both resistin and heparanase (active heparanase 0.6 µg/ml, 1.25 µg/ml, 5 µg/ml, **d–f** respectively). Each experiment was repeated 3 times yielding similar results. A representative experiment is presented. **e**. Quantification of cell differentiation (oil red staining) shown in each panel. Each data point represents the mean ±SE of triplicate wells. **p = 0.001 for resistin vs. resistin+heparanase (5 µg/ml); *p = 0.01 for resistin vs. resistin+heparanase (1.2 µg/ml).

THP1 human acute monocytic leukemia cells grow in suspension and in the presence of PMA are differentiated into adherent, macrophage-like foam cells characterized by the accumulation of oil droplets. Since resistin is known to enhance this process [25] we tested whether heparanase modulates this activity of resistin. Resistin augmented the ability of PMA to induce THP1 cell differentiation, in a dose-dependent manner (Figure 5). As little as 10 ng/ml of resistin already enhanced oil droplets accumulation compared to the treatment of cells with PMA alone, and further escalation in THP1 cell differentiation was readily observed at a concentration of 160 ng/ml. Cell differentiation and oil droplets were visualized and quantified by Oil red staining (Figure 5i; p = 0.001 for 20 vs. 160 ng/ml resistin). Addition of heparanase together with PMA did not facilitate THP1 cell differentiation (Figure 6A, panel b). However, the addition of latent heparanase together with resisitin (Figure 6A, panel d) markedly enhanced foam cell formation compared to resistin alone (Figure 6A, panel c). At a higher magnification (Figure 6B) the enhancement of oil droplets formation was readily observed (panel d), increase that is statistically highly significant (p = 0.001 for resistin vs. resistin+heparanase). A similar increase in THP 1 cell differentiation was obtained by combining active (GS3) heparanase with resistin (Figure 6C). Enhancement of foam cells formation was best noted with the highest tested concentration of active heparanase (5 µg/ml; p = 0.001 for resistin vs. resistin+heparanase) though eight fold lower concentration (0.6 µg/ml) was already effective (Figure 6C, panels f and d, respectively; p = 0.005). Taken together, our results indicate that resistin and heparanase form a complex and that heparanase enhances myeloid cell differentiation by resistin.

## Discussion

Combining an enriched source of natural proteins (500 fold concentrated normal human urine) together with a highly specific isolation method (affinity chromatography) enabled us the isolation and characterization of six bioactive soluble receptors, as well as enzymes and associated binding proteins (IL-18BP, IL-32BP/PR3) [21], [22]. No other approach would yield the latter. In a search for a heparanase receptor, postulated to mediate non-enzymatic functions of the heparanase protein [18], we utilized human urine as a source for heparanase-binding protein/soluble receptor(s). The affinity chromatography revealed the interaction of several urinary proteins with human heparanase (Figure 1) including the modulator resistin. Resistin is a 12.5 kDa cysteine-rich polypeptide discovered in a screen for adipocyte gene products that are down-regulated by anti-diabetic thiazolidinedione (TZD) drugs [26]. It is a member of a small family of secreted proteins characterized by a unique spacing of 10–11 cysteine residues known as resistin-like molecules (RELMs) [27] or FIZZ (found in inflammatory zone) proteins [28]. Accumulating data indicate that resistin, initially described as a rodent adipokine, is predominantly macrophage-derived in humans [29] and thought to link between inflammation, metabolic diseases and possibly tumorigenesis [28], [30]. In human macrophages, resistin has been shown to induce the production of inflammatory cytokines and to drive expression of cell adhesion molecules, including vascular cell adhesion molecule-1 (VCAM-1), inter cellular adhesion molecule-1 (ICAM-1) and monocyte chemoattractant protein-1 (MCP-1) [31], [32]. In vascular cells, resistin enhances vascular smooth muscle cell proliferation, endothelial dysfunction, and promotes monocyte adhesion and infiltration [28]. These functions involve the activation of p38 mitogen-activated protein kinase (MAPK), impaired insulin signaling, altered oxidative stress response and increased cell proliferation [33]. The mechanism by which resistin exerts its biological effects is largely obscure. The experiments described in this study point to a physical interaction between heparanase and resistin. The interaction was revealed by affinity chromatography (Figure 1), co-immunoprecipitation (Figure 2) and ELISA (Figure 3). Significantly, both latent and constitutively active heparanase proteins interacted with resistin and potentiated the differentiation of human monocytes to macrophages (Figure 6) [25]. Whether significant interaction between resistin and heparanase occurs at physiological conditions is yet to be discovered. Nevertheless, a marked increase in the level of both proteins was demonstrated under various pathological conditions (see below), thereby enabling complex formation. While demonstration of a seemingly high affinity interaction between resistin and heparanase (observed under stringent conditions) supports a functional consequence(s) such as enhancement of THP1 cell differentiation, the observed synergistic effect may be indirect and additional mechanistic studies (i.e., use of mutant proteins that fail to interact) are needed to prove a causal functionality for the heparanase-resistin complex.

The rationale for the observed unexpected association between heparanase and resistin may result from seemingly common features of the two proteins. Resistin is detected in human serum in nanogram concentrations [34], and is dramatically induced by inflammatory stimuli [32], [35], [36]. In a study of diabetic obese and lean subjects, serum resistin levels were elevated (∼10 fold) and strongly correlated with circulating markers of inflammation, including tumor necrosis factor (TNF)-α, and interleukin (IL)-6 [37]. Elevated resistin levels have also been shown in patients with rheumatoid arthritis (RA) and inflammatory bowel disease (IBD) [38]. Notably, an almost identical functional repertoire is reported for heparanase. Heparanase was shown to stimulate macrophage activation [16], which in colitis induces production (i.e., via TNFα) and activation (via cathepsin L) of latent heparanase in the colon epithelium, together generating a vicious cycle that powers colitis and the associated tumorigenesis [39]. Upregulation of heparanase was reported in different inflammatory and autoimmune conditions including, among others, delayed type hypersensitivity (DTH) [40], graft vs. host disease (GVHD) [41], inflammatory bowel disease (IBD) [39], rheumatoid arthritis (RA) [42], type 1 diabetes [43], atherosclerosis [16], [44] and sepsis [45]. Thus, it appears that both heparanase and resistin may play a common and co-operative role in inflammation and autoimmunity. The resistin receptor remains unknown, although recent reports suggested that resistin interacts with the endotoxin receptor Toll-like receptor-4 (TLR-4) and decorin [46], [47], [48]. Decorin has been shown to inhibit the expression of syndecan-1 [49], thus reducing syndecan-1-mediated cellular uptake and elevating the extracellular levels of heparanase [3]. Excess heparanase present extracellularly can potentially interact with resistin or modulate its interaction with decorin. It was discovered, however, that resistin interacts with an isoform of decorin that lacks proteoglycan (core protein) modification, and thus cannot associate with heparanase or serve as a substrate for HS cleavage [47]. Notably, TLR-4 mediates the pro-inflammatory function of heparanase in chronic inflammatory bowel conditions [50] and macrophage activation in atherosclerotic plaques [16]. The link of heparanase to inflammatory bowel disease is based on results obtained in a mouse model over-expressing heparanase, in which the inflammatory profile of macrophages was preserved for more than a month after termination of the pro-inflammatory DSS treatment [39]. It was further supported by *in vitro* observations employing mouse macrophages stimulated with LPS, a specific stimulator of TLR4 signaling [51], in which a marked increase in the induction of cytokines (e.g. TNFα, IL-6) known to be induced by TLR-4 signaling and to play important role in the pathogenesis of ulcerative colitis [39], [52], was observed. Similar to LPS, both TLR-4 and -2 appear to mediate the induction of cytokines in macrophages upon exposure to heparanase [16]. The same cytokines where shown to be induced by resistin in human adipocytes [53]. Taking into account the observed synergistic effect of resistin and heparanase in promoting THP1 cell differentiation, it is conceivable that a similar stimulatory and possibly synergistic effect may be noted in various inflammatory conditions known to be affected by heparanase and resistin. Human resistin is also a potential mediator of diabetes and cardiovascular disease. A European study of more than 20,000 healthy individuals found a higher risk for the development of myocardial infarction, stroke, coronary artery disease or impaired renal function in those in the highest quartile of resistin [38], [54], [55], [56]. This lies in accordance with increasing awareness of the significance of heparanase in atherosclerosis and heart diseases [44]. Heparanase is closely associated with development and progression of atherosclerotic plaques, including stable to unstable plaque transition [16], [44]. Consequently, heparanase levels are markedly increased in the plasma of patients with acute myocardial infarction [16]. Noteworthy, heparanase activates macrophages, resulting in marked induction of cytokine expression associated with plaque progression towards vulnerability [16]. Together, heparanase emerges as a regulator of vulnerable lesion development and potential target for therapeutic intervention in atherosclerosis and related vessel wall complications, possibly involving resistin. Heparanase is strongly implicated in cancer metastasis and neovascularization [2], [3], [4], [5], [9] and is up-regulated in essentially all human tumors examined [5]. Cancer patients exhibiting high levels of heparanase had a significantly shorter postoperative survival time than patients whose tumors contained low levels of heparanase due, in part, to increased tumor metastasis and tumor angiogenesis [5]. The association of resistin with cancer progression is less investigated and understood. Nevertheless, recent epidemiological studies have indicated that serum resistin is significantly elevated in patients with breast, gastric, colorectal, endometrial and esophageal carcinomas [57] clearly suggesting that heparanase and resistin are associated with the tumor and its microenvironment and can possibly co-operate and drive tumor progression. Studies aimed at this direction are currently in progress.
